# Isolation and Characterisation of 11 Polymorphic Microsatellite Markers in *Papaver rhoeas* L. (Corn Poppy), a Major Annual Plant Species from Cultivated Areas

**DOI:** 10.3390/ijms14010470

**Published:** 2012-12-24

**Authors:** Vaya Kati, Valérie Le Corre, Séverine Michel, Lydia Jaffrelo, Charles Poncet, Christophe Délye

**Affiliations:** 1 Weed Science Laboratory, Department of Pesticide Control & Phytopharmacy, Benaki Phytopathological Institute, 8 Stefanou Delta, Kifissia, 14561 Attiki, Greece; E-Mail: v.kati@bpi.gr; 2 INRA, UMR1347 Agroécologie, 17 rue Sully, F-21000 Dijon, France; E-Mails: lecorre@dijon.inra.fr (V.L.C.), severine.michel@dijon.inra.fr (S.M.); 3 INRA, UMR1095 Génétique, Diversité et Écophysiologie des Céréales, 234 avenue du Brézet, F-63100 Clermont-Ferrand, France; E-Mails: lydia.jaffrelo@clermont.inra.fr (L.J.), charles.poncet@clermont.inra.fr (C.P.)

**Keywords:** *Papaver rhoeas*, Papaveraceae, microsatellite markers, next-generation sequencing, genetic diversity

## Abstract

*Papaver rhoeas*, an annual plant species in the Papaveraceae family, is part of the biodiversity of agricultural ecosystems and also a noxious agronomic weed. We developed microsatellite markers to study the genetic diversity of *P. rhoeas*, using an enriched microsatellite library coupled with 454 next-generation sequencing. A total of 13,825 sequences were obtained that yielded 1795 microsatellite loci. After discarding loci with less than six repeats of the microsatellite motif, automated primer design was successful for 598 loci. We tested 74 of these loci for amplification with a total of 97 primer pairs. Thirty loci passed our tests and were subsequently tested for polymorphism using 384 *P. rhoeas* plants originating from 12 populations from France. Of the 30 loci, 11 showed reliable polymorphism not affected by the presence of null alleles. The number of alleles and the expected heterozygosity ranged from 3 to 7.4 and from 0.27 to 0.73, respectively. A low but significant genetic differentiation among populations was observed (*F*_ST_ = 0.04; *p* < 0.001). The 11 validated polymorphic microsatellite markers developed in this work will be useful in studies of genetic diversity and population structure of *P. rhoeas*, assisting in designing management strategies for the control or the conservation of this species.

## 1. Introduction

The *Papaver* genus in the Papaveraceae family comprises 100 species distributed in various countries around the world, from central and south Europe to temperate Asia, America, Oceania and South Africa [1]. *Papaver rhoeas* L. (corn poppy) is one of the most well-known members of this genus, easily identified by its scarlet flowers. This species has been the symbol for Remembrance Day (or Poppy Day) since the end of WWI, which makes it a heritage species. *P. rhoeas* is a semelparous species that thrives in disturbed land. It has a high reproductive potential through its seeds, which are dormant and have long viability, thus facilitating its persistence and spread [2–4]. Its abundant pollen is an important food source for honeybees during their foraging period [5] as well as for other beneficial insects [6,7]. On the other hand, *P. rhoeas* can dominate agricultural fields, where it is listed as a serious annual weed of winter cereal crops that can cause significant yield losses because of its high competitive ability [8]. The agronomic significance of *P. rhoeas* also includes indirect harmful effects on crops through the harbouring of phytopathogenic viruses [9].Various uses have been documented for all plant parts of *P. rhoeas* [10].

*P. rhoeas* is an insect-pollinated, diploid hermaphroditic species (2*n* = 14) with high self-incompatibility [11]. Outcrossing contributes to high levels of genetic variation and heterozygosity [12]. *P. rhoeas* has been reported to exhibit high phenotypic plasticity and high genetic variation, which for example results in variable petal pigmentation and leaf shape [13].

Quantification of genetic diversity within and among populations of *P. rhoeas* is important for the study of the ecology and population structure of this species. Such studies can assist in implementing management strategies in the context of either conservation ecology or weed control. Simple sequence repeat (SSR) markers, also called microsatellite markers, are popular tools used to assess the genetic diversity of species [14]. Their advantages include good repeatability, a high level of polymorphism and co-dominance. There are currently no microsatellite markers available for genetic comparisons between *P. rhoeas* populations. The aim of this work was to develop and validate polymorphic microsatellite markers for *P. rhoeas*, and to assess the genetic variation in 12 *P. rhoeas* populations from France.

## 2. Results and Discussion

### 2.1. Next-Generation Sequencing Results

A total of 13,825 sequences were obtained from the *P. rhoeas* enriched library. Average sequence length was 240 nucleotides, with a maximum length of 570 nucleotides. After discarding short sequences (less than 80 nucleotides) and sequences without microsatellite motifs, the remaining sequences were filtered for redundancy and multiple copies in the sequence data set. In total, 1795 validated microsatellite loci were identified. Automated primer design was successful for 829 loci. Loci with less than six repeats of the microsatellite motif were discarded, yielding 598 loci for which the QDD pipe-line generated 4453 primer pairs.

### 2.2. Development of Microsatellite Markers

We selected 74 of the 598 microsatellite loci based on the lowest value of the “penalty” index computed by QDD. A total of 97 primer pairs were tested to amplify the 74 loci. An amplicon was consistently obtained from 30 of the 74 selected loci. The 30 amplicons were sequenced in both directions with the corresponding primer pair. They were all confirmed to contain the expected microsatellite motif, and polymorphism was observed for all loci among the seven *P. rhoeas* plants used for screening. Among the 30 loci, 20 carried a dinucleotide motif and 10 a trinucleotide motif. The remaining 44 loci were not further considered because no amplicon could be obtained, or amplicons were not obtained for all seven *P. rhoeas* plants used for screening, or no polymorphism was detected after sequencing the amplicons.

The 30 microsatellite loci were further tested on 384 *P. rhoeas* plants (12 populations of 32 individuals each). The software MicroChecker [15] was used to identify microsatellites with null alleles or large allele drop-out (*i.e.*, short allele dominance due to PCR artefacts). There was no evidence for large allele drop-out in any loci. Null alleles can cause deviations from Hardy-Weinberg equilibrium, and can introduce substantial bias in population genetic analyses. Loci for which null alleles were detected in a majority of populations (more than seven populations among the 12 studied) were, therefore, not further considered. This screening resulted in a final set of 11 microsatellite loci, the characteristics of which are summarised in Table 1.

### 2.3. Genotyping and Population Genetics Analysis

A total of 384 plants from 12 natural populations of *P. rhoeas* collected in cultivated fields in France (32 plants per population) were genotyped at the 11 microsatellite loci. The average number of alleles per population (*N*_A_) was between 3.0 (locus PMS073) and 7.4 (locus PMS002). The mean expected heterozygosity per population (*H*_E_) also varied widely among loci, ranging from 0.271 (locus PMS073) to 0.731 (locus PMS037). Significant pairwise linkage disequilibrium was detected for only three pairs of loci: PMS005 and PMS073 (*p*-value < 0.01), PMS015 and PMS073 (*p*-value < 0.001) and PMS039 and PMS054 (*p*-value < 0.001).

A slight deficit in heterozygotes was observed for most loci in most populations, as indicated by positive *F*_IS_ values (Table 2). Locus PMS002 showed a significant deficit in heterozygotes in seven populations among the 12 studied. Microchecker analyses detected null alleles at this locus in these populations. This is likely the cause of the observed deviation from Hardy-Weinberg equilibrium. We therefore recommend this locus should be used with caution. Excluding locus PMS002 from the analyses did not alter the values of expected heterozygosity (*H*_E_) observed over all loci in each population. Four other loci (PMS015, PMS051, PMS052 and PMS061) also showed some deviation from Hardy-Weinberg equilibrium. However, for each locus, this was only observed in one or two populations among the 12 studied (Table 2).

Considering all loci except PMS002, the mean *F*_IS_ value within population varied between −0.005 and 0.14. Under the assumption that deviation from Hardy-Weinberg equilibrium was due solely to inbreeding, the rate of autogamy, calculated as *s* = 2 *F*_IS_/(1+ *F*_IS_), varied between 0% and 24% (overall mean: 11%). For several samples, the estimated rates of autogamy seemed to conflict with the self-incompatible mating system described in *P. rhoeas* [11]. We suspect that deviation from Hardy-Weinberg equilibrium in these samples resulted either from biparental inbreeding (*i.e.*, mating among relatives due to limited seed dispersal and restricted pollinator flight distances), or from population subdivision at the level of each sampled field (Walhund effect). This remains to be investigated.

**Figure 1:**
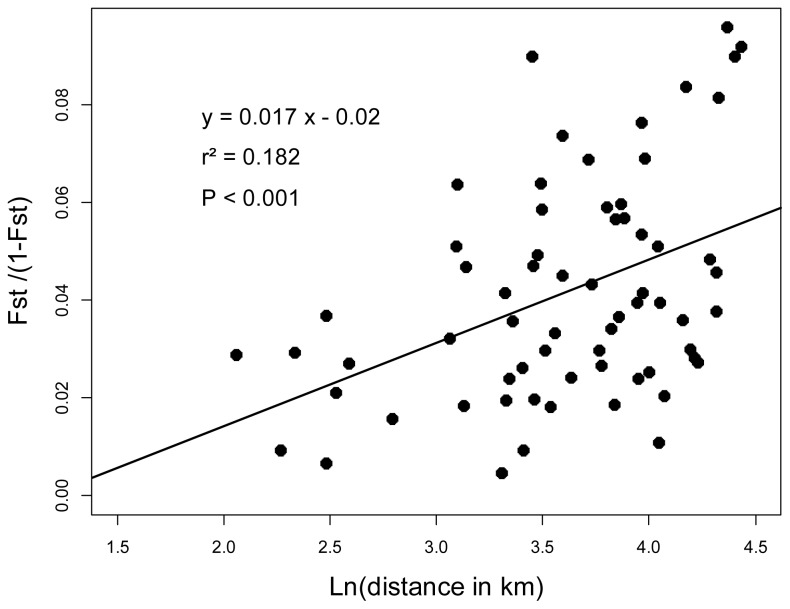
Plot of genetic distances versus geographic distances for all possible pairs of the sampled populations.

When considering all 11 loci, the expected genetic diversity within population was always high, ranging from 0.518 to 0.589 (overall mean: 0.547). The genetic differentiation among all populations, as measured by *F*_ST_, was 0.04. This indicated large population sizes and/or high gene flow, as expected in an allogamous plant species [12]. Although low, the genetic differentiation among populations was highly significant (*p*-value < 0.001). Pairwise *F*_ST_ values computed for all possible pairs of populations ranged from 0.009 to 0.087. A significant linear relationship was observed between pairwise *F*_ST_/(1 − *F*_ST_) values and the natural logarithm of geographical distances between populations (*r*^2^ = 0.182; *p* < 0.001) (Figure 1). This indicates isolation-by-distance among populations [16].

## 3. Experimental Section

### 3.1. Plant Material

Twelve *P. rhoeas* populations were sampled in agricultural fields in the Burgundy region (France) during the summer of 2011 (Table 3). Samples consisted of one green leaf collected from each of 32 plants per field. The plants sampled were distributed across the field.

### 3.2. Microsatellite Library and Primer Design

The approach used has been described elsewhere [17]. Briefly, a microsatellite-enriched library was generated using a high-throughput method based on the coupling of multiplex microsatellite enrichment and next-generation sequencing on a 454 GS-FLX Titanium platform. The sequences generated were analysed using the QDD pipe-line [18], following the procedure described in [17]. Primer pairs were designed automatically using the Primer3 algorithm [19] implemented in the QDD pipeline with the setup described in [17].

### 3.3. Loci Selection and Primer Editing

Microsatellite loci of interest were selected among those identified by running the QDD pipe-line using the following criteria: (1) a target microsatellite sequence containing at least six repeats of the microsatellite motif, and (2) an expected amplicon of 90 to 350 bp in length. Editing of the primer sequences generated by QDD was performed when necessary to ensure an annealing temperature around 60 °C, and the presence of a C or G nucleotide at each primer 3′ end to increase the stability of primer binding to its target. The edited primers were then checked for self-complementarity and complementarity between primers in a same pair to avoid the formation of primer dimers. Primers sequences were further edited as needed.

### 3.4. DNA Amplification and Genotyping

All primer pairs were tested for PCR amplification on DNA extracted from seven *P. rhoeas* plants originating from five populations: FE (3 plants), BE (1 plant), ST (1 plant), CS (1 plant) and PY (1 plant). Geographic origins of the populations are shown in Table 3. DNA was extracted using a rapid method [20]. PCR amplifications were performed using a Mastercycler (Eppendorf, Hamburg, Germany) thermocycler, in a 20 μL reaction mix containing 70 mM Tris-HCL, 2 mM MgCl_2_, 17 mM (NH_4_)_2_SO_4_, 10 mM beta-mercaptoethanol, 0.05% (wt/vol) polyoxyethylene-ether W1, 0.2 mg/mL bovine serum albumin, 200 mM of each dNTP, 10 ng genomic DNA, 0.5 units of Taq DNA polymerase, and 0.2 μM each of reverse and forward primers. The PCR program used consisted of 5 min at 95 °C, followed by 37 cycles of 5 s at 95 °C, 10 s at 60 °C and 30 s at 72 °C [21]. Amplicons were visualised under UV light by electrophoresis on a 3% (wt/vol) agarose gel stained with ethidium bromide. When an intense amplicon was obtained from all seven plants, it was sequenced in both directions by Sanger sequencing to confirm the presence of the expected microsatellite motif. Primer pairs yielding amplicons in which the presence of the expected microsatellite motif was confirmed and polymorphism was observed by sequencing were used to genotype the corresponding microsatellite locus in the 12 *P. rhoeas* populations of 32 individuals each (Table 3).

The DNA extracts of all plants were diluted 50-fold prior to genotyping with fluorescent labelled markers. PCR products were dye-labelled (6-FAM, NED, VIC or PET directly attached to the forward primer in each primer pair) and assayed on an ABI 3730XL sequencer (Applied Biosystems, Foster City, CA, USA) using 500 liz (Applied Biosystems, Foster City, CA, USA) as a size standard. Amplicon sizes were analyzed with GeneMapper 4.0 (Applied Biosystems, Foster City, CA, USA).

### 3.5. Data Analysis

Micro-Checker v2.2.3 [15] was used to detect genotyping errors due to null alleles, stuttering, or allele dropout. The FSTAT software [22] was used to estimate standard genetic diversity parameters: the number of alleles per locus (*N*_A_), observed (*H*_O_) and expected (*H*_E_) levels of heterozygosity as well as the inbreeding coefficient *F*_IS_ were estimated for each population. Genetic differentiation among populations was estimated using Weir and Cockerham’s *F*_ST_ [23]. Deviations from Hardy-Weinberg equilibrium (HWE) at each locus within each sample were tested in FSTAT using 5000 random permutations of alleles among individuals. Because of multiple testing within each sample, we used a nominal significance level of 0.01. Significance of the genetic differentiation among populations as measured by *F*_ST_ was tested, based on 5000 random permutations of genotypes among populations.

## 4. Conclusions

The 11 polymorphic microsatellites developed for *P. rhoeas* proved reliable for population genetic studies. Based on a sample of populations clustered within roughly 150 km, we observed a level of genetic differentiation that was low but that increased significantly with geographical distances. This suggests that addressing population structure in *P. rhoeas* at a geographical scale larger than the scale considered here should reveal more genetic polymorphism and higher levels of genetic differentiation. On the other hand, fine-scale studies are necessary to elucidate the existence of some genetic structure within cultivated fields. Future studies, based on the 11 polymorphic microsatellites we developed, will enable a better understanding of the patterns of genetic diversity of *P. rhoeas*, providing information on population structure and dynamics that can be useful for guiding management strategies.

## Figures and Tables

**Table 1:** The set of 11 microsatellite markers developed in *P. rhoeas*. F, forward, and R, reverse primer sequences, *T*_a_, annealing temperature.

Locus	Repeat motif	Primer sequences (5′-3′)	*T*_a_ (°C)	Size range (bp)	GenBank accession No.
PMS002	TC	F: TTCACAACCTAAGTTCCCCTG	60	107–139	HF547289
		R: ACAAATCGAAACCCCTAATTTG	60		
PMS005	CT	F: CCCCAATCAAAGAAGCTTGATG	60	170–183	HF547290
		R: GATATGATATGTCCCTCTCAATGG	60		
PMS006	GA	F: GAATTCCATTCCCACTCAATATC	60	241–255	HF547291
		R: CAGCAGCAGCATTTATCCTCAAC	60		
PMS015	AC	F: ATCCCCTGTTGATCCAATTG	60	203–222	HF547292
		R: TTGCGATGTTTATAGGGCAC	60		
PMS037	TCT	F: ACTGATACTACTTCTTCCTCCACC	60	78–193	HF547293
		R: TCGAAGAGCCTGTATTTGAATC	60		
PMS039	TC	F: TTGATCTGCTCTTACAAACCC	60	105–123	HF547294
		R: CCAGAAAAGTAGAATATTGATTGAGTTG	60		
PMS051	GAA	F: GGAATCTCGTGGCATTCATTTAC	60	201–241	HF547295
		R: GAATCTTCTCCAAACACATCGAAC	60		
PMS052	AG	F: TAGCTGTACGGAAGAGCAAGC	60	168–249	HF547296
		R: CGATCTCTTCCCGTGTCC	60		
PMS054	CA	F: GACTTAAACTCGGCAACATCAC	60	51–153	HF547297
		R: ATATGGTTGTGAATGAGTTAGCTTG	60		
PMS061	TG	F: GATGTGCGTCTACGAGATTTGG	60	211–221	HF547298
		R: ACCGATTACCAGAAACAGATCG	60		
PMS073	TTC	F: TCTTCTGCATAAGGAGCATGAG	60	127–145	HF547299
		R: TGATGATATCTTGGAAGAATTGG	60		

**Table 2:** Results of the initial primer screening on 32 individuals from 12 populations of *P. rhoeas*.

Locus	BE				CF				CY			

	*N*_a_	*H*_O_	*H*_E_	*F*_IS_	*N*_a_	*H*_O_	*H_E_*	*F*_IS_	*N*_a_	*H*_O_	*H*_E_	*F*_IS_
PMS002	9	0.548	0.808	0.321 ^*^	6	0.625	0.723	0.136	10	0.366	0.607	0.396 ^*^
PMS005	6	0.594	0.700	0.152	6	0.687	0.690	0.003	9	0.687	0.769	0.106
PMS006	5	0.562	0.641	0.122	5	0.719	0.775	0.073	5	0.742	0.709	−0.046
PMS015	7	0.437	0.485	0.098	4	0.500	0.592	0.156	7	0.406	0.719	0.435 ^*^
PMS037	6	0.687	0.730	0.058	4	0.594	0.671	0.116	9	0.656	0.813	0.193
PMS039	6	0.750	0.776	0.034	5	0.594	0.739	0.196	7	0.594	0.688	0.136
PMS051	4	0.312	0.332	0.058	3	0.156	0.148	−0.054	6	0.344	0.308	−0.116
PMS052	4	0.129	0.336	0.616 ^*^	5	0.125	0.179	0.303	8	0.531	0.616	0.138
PMS054	5	0.531	0.568	0.065	6	0.625	0.583	−0.072	5	0.500	0.406	−0.232
PMS061	4	0.344	0.327	−0.052	4	0.406	0.442	0.080	2	0.344	0.425	0.192
PMS073	3	0.156	0.149	−0.047	3	0.500	0.411	−0.216	4	0.500	0.415	−0.205

**Locus**	**CS**				**DN**				**FE**			

	***N*_A_**	***H*_O_**	***H*_E_**	***F*_IS_**	***N*_A_**	***H*_O_**	***H*_E_**	***F*_IS_**	***N*_A_**	***H*_O_**	***H*_E_**	***F*_IS_**

PMS002	4	0.166	0.217	0.231	6	0.333	0.763	0.563 ^*^	7	0.387	0.419	0.076
PMS005	5	0.625	0.662	0.056	4	0.687	0.638	−0.078	5	0.677	0.688	0.016
PMS006	5	0.562	0.709	0.207	5	0.625	0.698	0.105	6	0.656	0.661	0.007
PMS015	5	0.656	0.722	0.091	5	0.437	0.452	0.032	3	0.594	0.513	−0.158
PMS037	8	0.750	0.758	0.010	5	0.594	0.741	0.199	6	0.719	0.692	−0.039
PMS039	7	0.774	0.817	0.053	6	0.625	0.638	0.021	5	0.719	0.716	−0.004
PMS051	5	0.562	0.461	−0.220	8	0.344	0.436	0.212	6	0.313	0.404	0.227
PMS052	4	0.500	0.405	−0.235	6	0.281	0.335	0.161	5	0.290	0.338	0.140
PMS054	7	0.719	0.605	−0.187	6	0.625	0.656	0.048	3	0.581	0.475	−0.223
PMS061	4	0.375	0.424	0.116	3	0.531	0.458	−0.161	4	0.500	0.513	0.026
PMS073	3	0.344	0.297	−0.156	3	0.313	0.280	−0.117	3	0.313	0.279	−0.119

**Locus**	**IS**				**MA**				**MI**			

	***N*_A_**	***H*_O_**	***H*_E_**	***F*_IS_**	***N*_A_**	***H*_O_**	***H*_E_**	***F*_IS_**	***N*_A_**	***H*_O_**	***H*_E_**	***F*_IS_**

PMS002	7	0.500	0.793	0.369 ^*^	7	0.400	0.623	0.358 ^*^	9	0.516	0.719	0.283 ^*^
PMS005	6	0.844	0.751	−0.124	5	0.500	0.679	0.264	5	0.600	0.484	−0.240
PMS006	5	0.594	0.728	0.184	5	0.625	0.658	0.050	5	0.594	0.718	0.173
PMS015	6	0.406	0.425	0.045	5	0.500	0.624	0.199	5	0.531	0.584	0.091
PMS037	5	0.594	0.690	0.139	6	0.781	0.741	−0.054	5	0.531	0.652	0.185
PMS039	6	0.906	0.788	−0.150	6	0.656	0.616	−0.065	5	0.719	0.696	−0.033
PMS051	5	0.156	0.207	0.246	6	0.250	0.475	0.474 ^*^	7	0.438	0.490	0.108
PMS052	5	0.625	0.561	−0.114	4	0.219	0.303	0.279	6	0.226	0.267	0.153
PMS054	4	0.406	0.505	0.196	6	0.531	0.592	0.103	4	0.281	0.426	0.340
PMS061	4	0.188	0.329	0.430 ^*^	3	0.219	0.352	0.378	4	0.484	0.455	−0.064
PMS073	2	0.156	0.146	−0.069	3	0.406	0.343	−0.185	3	0.344	0.303	−0.133

**Locus**	**PY**				**ST**				**TY**			

	***N*_A_**	***H*_O_**	***H*_E_**	***F*_IS_**	***N*_A_**	***H*_O_**	***H*_E_**	***F*_IS_**	***N*_A_**	***H*_O_**	***H*_E_**	***F*_IS_**

PMS002	9	0.433	0.559	0.225	8	0.467	0.764	0.389 ^*^	7	0.531	0.665	0.201
PMS005	5	0.563	0.715	0.213	5	0.656	0.600	−0.094	5	0.375	0.526	0.287
PMS006	6	0.719	0.729	0.014	4	0.656	0.649	−0.012	4	0.839	0.682	−0.229
PMS015	7	0.688	0.662	−0.038	6	0.469	0.429	−0.092	5	0.531	0.592	0.103
PMS037	9	0.750	0.771	0.027	7	0.719	0.804	0.106	5	0.688	0.713	0.079
PMS039	6	0.656	0.780	0.158	6	0.719	0.718	−0.001	7	0.656	0.688	0.045
PMS051	4	0.281	0.257	−0.096	4	0.125	0.348	0.641 ^*^	7	0.469	0.504	0.069
PMS052	5	0.344	0.458	0.249	5	0.387	0.477	0.188	8	0.469	0.447	−0.050
PMS054	7	0.613	0.562	−0.091	6	0.531	0.643	0.174	6	0.563	0.621	0.095
PMS061	4	0.400	0.542	0.262	3	0.469	0.487	0.037	6	0.290	0.341	0.150
PMS073	3	0.219	0.203	−0.080	3	0.281	0.252	−0.116	3	0.188	0.176	−0.063

*N*_A_: number of alleles, *H*_O_: observed heterozygosity, *H*_E_: expected heterozygosity, *F*_IS_: inbreding coefficient. (^*^) indicates significant deviation from Hardy-Weinberg equilibrium (*p*-value < 0.01).

**Table 3:** Geographic origins of the 12 *P. rhoeas* populations used in this study.

Code	Population	Latitude (N)	Longtitude (E)	Altitude (m)
BE	Beaunotte	47.678028	4.681338	302
CF	Chamboeuf	47.234224	4.890333	471
CY	Chevigny	47.181531	5.475732	215
CS	Chivres	46.977046	5.102653	175
DN	Détain	47.155948	4.782923	585
FE	Fénay	47.233031	5.065271	221
IS	Isômes	47.643463	5.284272	277
MA	Marliens	47.218520	5.190730	196
MI	Mirande	47.302668	5.080832	245
PY	Perrigny	47.287745	5.453528	190
ST	St Thibault	47.368827	4.472117	356
TY	Talmay	47.380004	5.462555	217
